# Evidence That Gene Activation and Silencing during Stem Cell Differentiation Requires a Transcriptionally Paused Intermediate State

**DOI:** 10.1371/journal.pone.0022416

**Published:** 2011-08-19

**Authors:** Jonathan L. Golob, Roshan M. Kumar, Matthew G. Guenther, Lil M. Pabon, Gabriel A. Pratt, Jeanne F. Loring, Louise C. Laurent, Richard A. Young, Charles E. Murry

**Affiliations:** 1 Departments of Pathology and Bioengineering, Center for Cardiovascular Biology, Institute for Stem Cell and Regenerative Medicine, University of Washington, Seattle, Washington, United States of America; 2 Whitehead Institute for Biomedical Research, Cambridge, Massachusetts, United States of America; 3 Center for Regenerative Medicine, The Scripps Research Institute, La Jolla, California, United States of America; 4 Department of Reproductive Medicine, University of California San Diego, San Diego, California, United States of America; Instituto Nacional de Câncer, Brazil

## Abstract

A surprising portion of both mammalian and Drosophila genomes are transcriptionally paused, undergoing initiation without elongation. We tested the hypothesis that transcriptional pausing is an obligate transition state between definitive activation and silencing as human embryonic stem cells (hESCs) change state from pluripotency to mesoderm. Chromatin immunoprecipitation for trimethyl lysine 4 on histone H3 (ChIP-Chip) was used to analyze transcriptional initiation, and 3′ transcript arrays were used to determine transcript elongation. Pluripotent and mesodermal cells had equivalent fractions of the genome in active and paused transcriptional states (∼48% each), with ∼4% definitively silenced (neither initiation nor elongation). Differentiation to mesoderm changed the transcriptional state of 12% of the genome, with roughly equal numbers of genes moving toward activation or silencing. Interestingly, almost all loci (98–99%) changing transcriptional state do so either by entering or exiting the paused state. A majority of these transitions involve either loss of initiation, as genes specifying alternate lineages are archived, or gain of initiation, in anticipation of future full-length expression. The addition of chromatin dynamics permitted much earlier predictions of final cell fate compared to sole use of conventional transcript arrays. These findings indicate that the paused state may be the major transition state for genes changing expression during differentiation, and implicate control of transcriptional elongation as a key checkpoint in lineage specification.

## Introduction

Transcriptional pausing is the phenomenon in which genes experience initiation of transcription without elongation. Initially thought to occur only in rare instances such as Drosophila heat shock genes (reviewed in [1]), Krumm et al proposed that pausing might be a more general mechanism based on detailed analysis of the mouse *c-myc* gene [2]. More recent studied using chromatin immunoprecipitation followed by chip hybridization (ChIP-chip) or deep sequencing (ChIP-seq) has revealed that transcriptional pausing does, indeed, occur at a large fraction of genes in mammalian [1], [3], [4], [5], [6], [7] and Drosophila [8], [9] cells. Non-productive short transcripts in both the sense and antisense [10], [11] directions have been described at many genes, and many developmental regulatory genes appear to be paused in both pluripotent and differentiated cell types. In contrast, constitutively active housekeeping genes generally do not exhibit transcriptional pausing [12]. This suggests that transcriptional pausing is a widespread mechanism of controlling cell-type specific gene expression programs.

Despite these advances, the physiological significance of transcriptional pausing is still unclear. We noted that most studies of pausing, to date, have focused on cells in the steady state. We hypothesized that pausing may be important for permitting cells to rapidly change gene expression levels in response to environmental cues. The differentiation of pluripotent stem cells towards more committed cells provides a human model system to study one of the most dramatic changes of cellular states. We sought to understand how the transcription apparatus is dynamically regulated as new patterns of gene expression are established during differentiation. To test the hypothesis that transcriptional pausing is a key transition state for gene expression, we used a directed system to differentiate human embryonic stem cells to early mesoderm [13] and quantified the flow of protein-coding loci between active, paused and silent states using a combination of chromatin immunoprecipitation and 3′ transcript analysis.

## Materials and Methods

### Human Embryonic Stem Cell Culture

**Figure 1:**
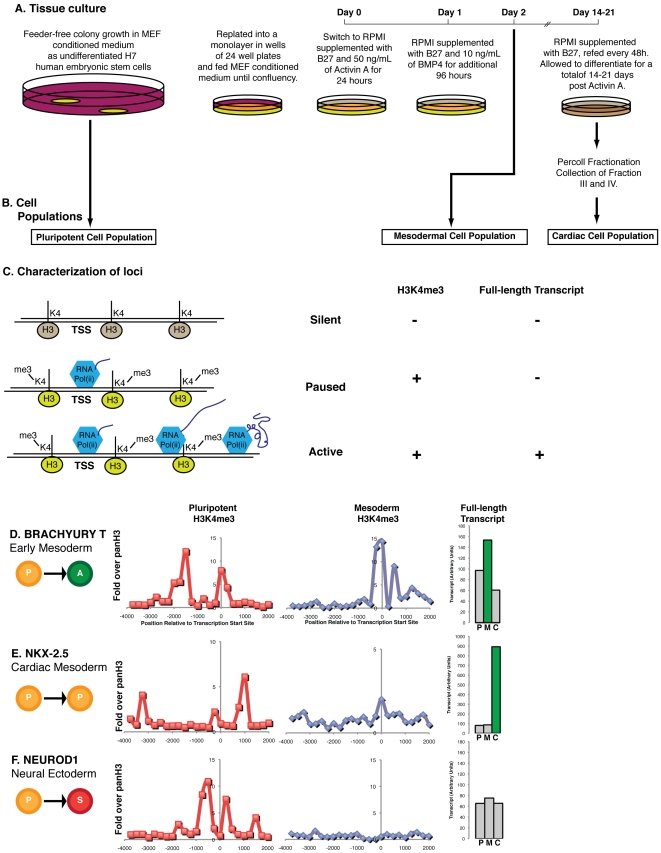
Characterization of the transcriptional state of developmentally regulated loci with known functions. (**A**) H7 human embryonic stem cells (WiCell) were maintained in MEF conditioned medium [35], replated into a high density monolayer in wells of a 24-well plate and differentiated as previously described in RPMI supplemented with B27 with the sequential addition of Activin A or BMP4. (**B**) The pluripotent cell population was collected from the undifferentiated human embryonic stem cells in colony growth conditions, the mesodermal population collected 48 hours after the addition of Activin A and the cardiac population was collected from cells 14–21 days after Activin A addition and enrichment via a Percoll density gradient purification [36]. (**C**) Loci were characterized as being silent if they lacked both tri-methylation on lysine 4 of histone H3 and full-length transcript, paused if they had detectable tri-methylation on lysine 4 of histone H3 only, or active if they had both full-length transcript and tri-methylation of lysine 4 on histone H3. (**D**) The *BRACHYURY T* locus, essential for mesoderm formation, is paused (P) in pluripotency and active (A) in mesoderm, is bound by tri-methyl lysine 4 on histone H3 (H3K4me3) in both pluripotency and mesodermal cell populations, with full length transcript above our background threshold (green, non-significant transcript in grey) only in the mesodermal (M) population, not in pluripotency (P) or cardiomyocytes (C). (**E**) The *NKX-2.5* locus, encoding a transcription factor essential for cardiomyocyte differentiation, is paused in both early populations, is modified with H3K4me3 in both pluripotent and mesodermal populations of cells, with transcription above our background threshold only in the cardiac cell population. (**F**) The *NEUROD1* locus, encoding for a transcription factor essential for neuronal ectoderm, is paused in the pluripotent population, becomes silent in the mesodermal population, is associated with H3K4me3 in the pluripotent but not mesodermal population of cells, and does not produce full-length transcript above a background threshold in any observed population of this cardiac mesoderm directed differentiation.

Undifferentiated H7 (WiCell) human embryonic stem cells were maintained on Matrigel-coated plates in mouse embryo fibroblast (MEF) conditioned medium as previously described [14], [15], [16]. For directed differentiation, cells were replated on Matrigel into a twenty-four well plates at an initial density of 200,000 cells per well, grown to a confluent monolayer in conditioned medium and sequentially exposed to 50 ng/mL Activin A on day 0 and 10 ng/mL of BMP4 on day one (both from R&D) in RPMI supplemented with B27 (Invitrogen) as previously described [17]. Cells were subsequently fed every other day with RPMI supplemented with B27 until collection. (Fig. 1A). Mesodermal cells were harvested on day 2 (the peak of *BRACHYURY T* expression) and beating cardiomyocyte cultures were collected on day 14 [18]. Only differentiations resulting in a strong induction of *BRACHYURY T* but not *SOX1* transcription at day 2 were subjected to further analysis.

### Chromatin Immunoprecipitation

Samples were prepared and analyzed as recently described by Nelson et al. [19], [20]. In brief, cells were cross-linked for 15 minutes at room temperature with 1.42% final concentration of formaldehyde. The cross-linking was quenched by the addition to 125 mM glycine, and cells were scraped off the plates. Cells were centrifuged (2000×*g* for 5 minutes) and washed twice with cold PBS and stored at −80°C. Thawed pellets were lysed with IP buffer (150 mM NaCl, 50 mM Tris-HCl pH 7.5, 5 mM EDTA, 0.5% v/v NP-40, 1.0% v/v Triton X-100 in deionized water) supplemented with Complete™ Protease Inhibitors Cocktail Tablets (Roche). The nuclear pellet was collected after centrifugation (12,000×*g* for 10 minutes), resuspended in IP buffer and sonicated for 6 rounds of 10 one-second pulses with half-second pauses at a power level of 5 (Misonix 3000 sonicator with a micro tip Cat#S3000). Sheared chromatin was distributed such that approximately 2×10^6^ cells-worth was combined with an antibody recognizing tri-methyl lysine 4 on histone H3 (H3K4me3) or pan-histone H3. Protein-A sepharose beads (Amersham Cat#17-5280-01) were used to collect precipitated nucleosomes. After extensive washes with IP buffer, 100 µL of 10% w/v Chelex® 100 Molecular Biology Grade Resin (Bio-Rad Cat#142-1253) beads were added to each sample, and the samples were boiled for 10 minutes after a brief vortex. Proteinase K (Invitrogen Cat# 25530-049, 1 µL of 20 microgram per milliliter) was added and samples were digested 30 minutes at 55°C at 1200 RPM in a shaking heat block, and then boiled for an additional 10 minutes.

### Promoter Array Hybridization

Immunoprecipitated H3K4me3 DNA and control pan-histone H3 immunoprecipitated DNA were purified by treatment with RNAse A, proteinase K and two successive phenol∶chloroform∶isoamyl alcohol extractions. Purified DNA ends were blunted using T4 DNA polymerase, and were then ligated to linker oligonucleotides. The resulting DNA was amplified using a two-stage ligation-mediated PCR (LMPCR) protocol. Amplified DNA was labeled with either Cy5-dUTP (H3K4me3) or Cy3-dUTP (histone H3) using Bioprime random primer labeling kits (Invitrogen) and purified using spin columns. Labeled DNA was then mixed (5–6 µg each of immunoenriched H3K4me3 DNA and control histone H3 DNA) and hybridized to arrays in Agilent hybridization chambers for up to 40 hours at 65°C. Arrays were then washed and scanned in an Agilent DNA microarray scanner BA using the extended dynamic range setting. Complete details of the entire ChIP-chip procedure are described in [21].

The human promoter array (244K Human Promoter Array) was purchased from Agilent Technology (www.agilent.com). The array consists of 2 slides each containing ∼244,000 60mer oligos designed to cover regions between approximately −4 kb and +4 kb relative to the transcription start sites of ∼18,464 genes at a density of about 1 oligo per 250 bp.

### Transcriptional Profiling

Each sample was prepared from approximately 1×10^6^ cultured cells. Two biological replicates were analyzed for each condition (pluripotent, mesoderm, and cardiomyocyte). Total RNA was purified the MirVana RNA purification kit (Invitrogen, Carlsbad, CA). Sample amplification, labeling and hybridization on Illumina Human WG6 Sentrix BeadChips were performed for all arrays in this study according to the manufacturer's instructions (Illumina, Inc., San Diego, CA) using an Illumina BeadStation in the Burnham Institute's Microarray Core Facility. Oligo dT priming ensured that only full-length transcription was measured. Raw data extraction was performed in the BeadStudio software suite (Illumina, Inc., San Diego, CA) and probes with a detection score less than 0.99, a measure of confidence that the signal observed is above background fluorescence, in all of the samples were discarded. The resulting probes were then quantile-normalized to correct for between-sample variation [22], [23].

### Determination of Full Length Transcription

If the detection *p*-value for a given transcript was less than 10^−3^ (as compared to the fluorescent intensity of negative control probes, in a two-sample Student's t-test) we considered the locus to be producing full-length transcript.

### Determination of Histone H3 Lysine 4 tri-methylation

H3K4me3 data were normalized and stored in metagene format as log2 over panH3. If any of the following are true, then the locus is determined to be H3K4me3 bound (Fig. S1A):

- Any point over 1.5 log2 over panH3.
- Any point over 0.8 log2 over panH3 within −1250 to 750 bp relative to the transcription start site

### Quantification of Transcriptional State Transitions

A customized, Python-based program was written to assign loci to specific transcription states and characterize the transitions between these states. For each cell population, if a locus is called as having both full-length transcript and H3K4me3, it is placed into a list of actively transcribed loci. If the locus instead is only called as being H3K4me3 bound, it is placed in the paused list. If the locus is neither called for having full-length transcript nor H3K4me3, it is added in the silent list (Fig. S1B).

For two cell populations and three possible transcriptional states, there are nine potential transitions. Using the lists of transcriptionally active, paused or silent loci for the pluripotent and mesodermal cell populations generated, we employed the following logic (Fig. S1C). If a locus starts in the transcriptionally active list in the pluripotent cell population, we look for it in the active, paused or silent list in mesoderm and then add it to the appropriate transition list. This process is repeated for loci starting as paused and silent.

### Gene Ontology Analysis

For gene ontology analysis, we used NCBI's capture of the gene ontology database in the gene2go.gz file, to gather the list of loci annotated as being within a given ontology. For each of these loci, we determine which of the nine possible transitions the locus is undergoing using the genome-wide lists previously generated.

## Results

In order to assess the relative contribution of the paused state to early human development, we used a recently established system of directing the differentiation of human embryonic stem cells to early mesoderm [20]. Briefly, Activin A and BMP4 were used to direct pluripotent H7 human embryonic stem cells to a mesoderm, or primitive-streak-like population, within 48 hours of growth factor addition (Fig. 1A). This differentiation protocol provides two distinct stages of early embryonic differentiation (pluripotent and mesoderm)[20], separated by a short enough time to allow us to directly observe the transcriptional activation or silencing of developmentally regulated loci (Fig. 1B).

We used the histone H3-lysine-4 trimethyl modification (H3K4me3) as a chromatin marker of transcriptional initiation [3]. Chromatin immunoprecipitates containing H3K4me3 were used to probe promoter arrays to globally define 5′ initiation. Gene expression microarrays were used to measure 3′ mRNA transcript, a result of productive transcriptional elongation. Using these measurements, we classified genes into distinct transcriptional states: active (both initiating and elongating transcription; paused (transcriptionally initiating but not elongating); and silent (not transcriptionally initiating) (Fig. 1C). To provide an end-point for the differentiation process, the mesoderm derivatives were further matured to beating cardiac myocytes, and full-length transcription was determined in this definitive stage.

We first focused on three developmentally regulated loci with known functions in lineage commitment to see that they behaved as expected in our differentiation system: BRACHYURY T (a transcription factor required for mesoderm differentiation)[24], NKX-2.5 (a transcription factor critical for cardiomyocyte differentiation)[25] and NEUROD1 (a transcription factor required for neuronal cell differentiation)[26]. In the pluripotent state the BRACHYURY T locus contained extensive H3K4me3 but produced minimal full-length transcript, indicating the locus was paused in embryonic stem cells. After differentiation to mesoderm, BRACHYURY T retained H3K4me3 binding, and increased levels of full-length transcript were detected, indicating a switch to active transcription (Fig. 1D). The cardiac transcription factor NKX-2.5 was associated with H3K4me3 but did not produce significant full-length transcript in either pluripotent or mesodermal populations, indicating it was transcriptionally paused in both stages (Fig. 1E). In contrast, NKX-2.5 showed high levels of 3′ transcript in definitive cardiomyocytes at two weeks. The NEUROD1 locus did not produce significant amounts of full-length transcript in any of the populations observed (Fig. 1F). The NEUROD1 locus was associated with H3K4me3 in embryonic stem cells and had a marked loss of H3K4me3 in mesoderm. This indicates that NEUROD1 transitions from being paused in embryonic stem cells to silent in mesodermal cells. This transcriptional silencing of NEUROD1 is consistent with lineage commitment away from ectodermal derivatives, like neurons, during the induction of mesoderm. Suppression of ectoderm is also supported by the absence of 3′ transcript for the ectodermal transcription factor, SOX1, during our differentiation protocol (data not shown).

**Figure 2:**
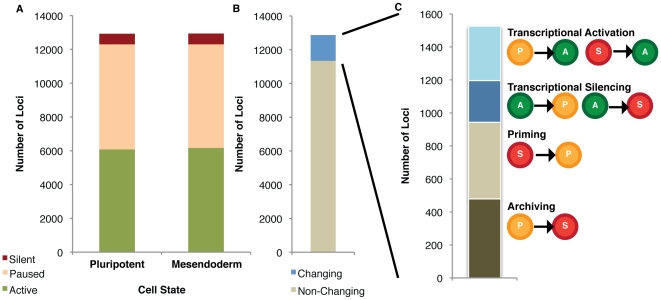
Categorization of protein-coding loci in the human genome into active (A), paused (P) or silent (S) states in pluripotency and mesoderm. (A) Overall distribution of loci among the three states is similar in each cell population. (B) Upon differentiation to mesoderm, 11.9%–18.0% of loci (1526–2108 genes; 2 independent experiments with representative figure shown) changed from one state to another. (C) Of those loci changing state, 14.0% to 21.6% (297–330 loci) underwent transcriptional activation from non-transcribed or initiating-only to productive transcription, 12.5 to 16.6% (253–264 loci) underwent transcriptional silencing from productive to initation-only or non-transcribed, 25.2% to 30.4% (464–532 loci) underwent priming from non-transcribed to initiation-only, and 31.4% to 48.1% (479–1015 loci) were archived from initiation-only to non-transcribed.

Having verified that genes with well-established biological functions behave as expected in our system, we next computationally sorted all detectable protein-coding loci in the human genome into one of the three transcriptional states (active, paused or silent) based on their association with H3K4me3 and full-length transcript abundance (Fig. 2A). We then determined how many loci were changing between these three states during differentiation from embryonic stem cells to mesoderm (Fig. 2B). Interestingly, the overall distribution of the 12,867 analyzed protein-coding loci among active (47% in pluripotency, 47.7% in mesoderm), paused (48.1% in pluripotency, 47.4% in mesoderm) and silent (4.8% in pluripotency, 4.9% in mesoderm) states did not change significantly during the transition from embryonic stem cells to mesoderm (Fig. 2A). There were many offsetting changes, however, with 1526 loci (11.9%) changing state (Fig. 2B). Of those loci that change transcriptional state, most are either “priming” from silent to paused (30.4% of the changing loci), or “archiving” from paused to silent (31.4% of changing loci) (Fig. 2C).

**Figure 3:**
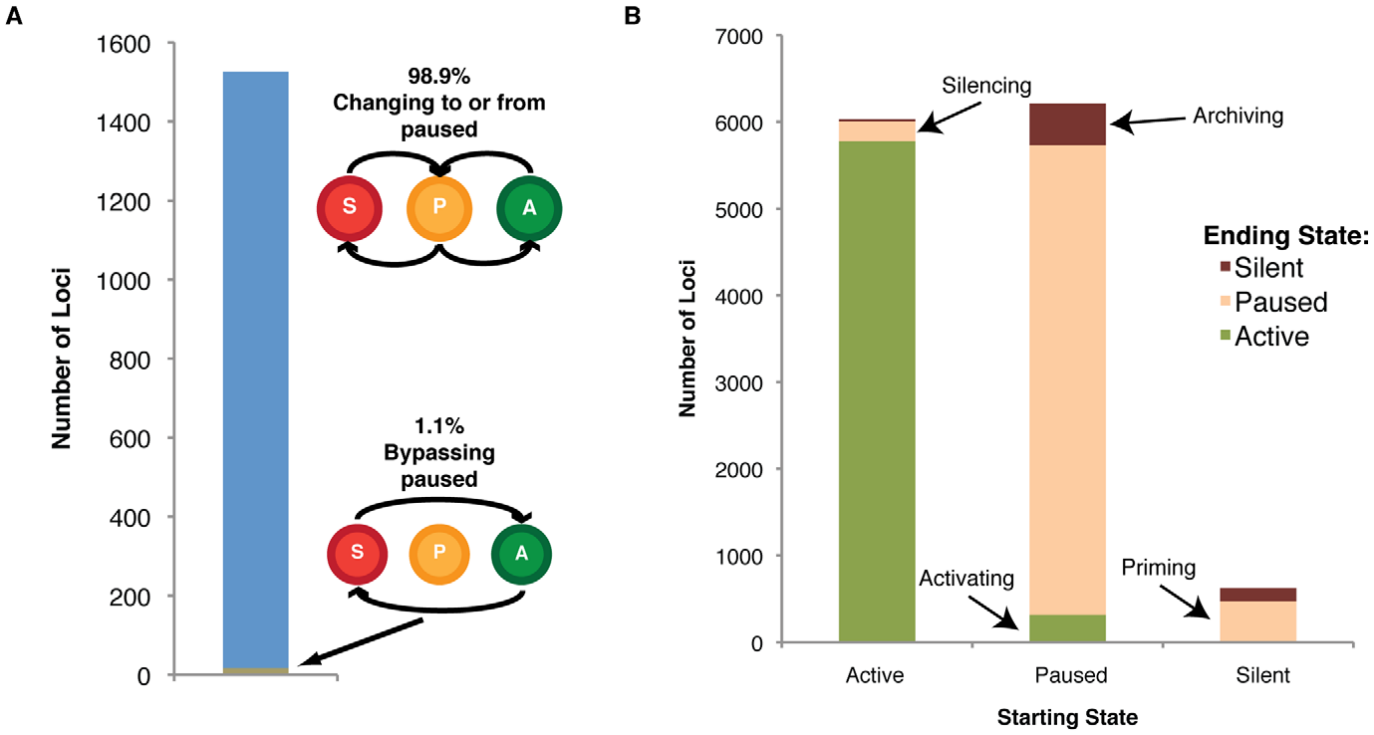
The vast majority of genes changing state proceed through a paused intermediate. (A) The overwhelming majority of loci changing state move either into or out from the paused state (P) (98.0 to 98.9% of changing loci) rather than directly from active transcription (A) to silent (S) or vice versa (1.1% to 2.0% of changing loci), strongly indicating that this paused state is a crucial waypoint in the control of developmentally regulated loci. (B) In the transition from pluripotency to mesoderm, the majority of loci stayed in the same state, with smaller portions silencing from active to paused, priming from silent to paused, transcriptionally activating from paused to active transcription or archiving from paused to silent. Only a few loci directly transitioned from active transcription to silent or silent to active transcription, bypassing the initiation-only step; these could be events not captured due to limits of temporal resolution.

We next sought to determine if genes changing expression transition directly from an active to a silent state (or vice-versa), or if they instead pass through a paused intermediate. Strikingly, we found an overwhelming 98.0 to 98.9% of genes changing expression are transitioning into or out from a paused intermediate (Fig. 3A). These data indicate that the paused transcriptional state is a crucial control waypoint for developmentally regulated loci, and that initiation and elongation are distinctly regulated in hESC differentiation.

**Figure 4:**
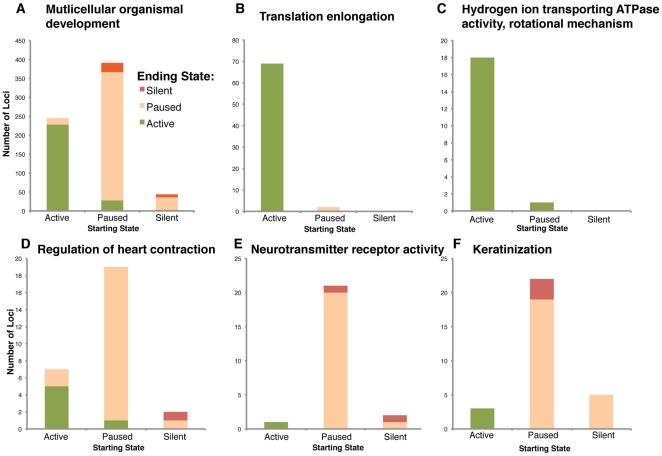
Transcriptional state transition of loci in various gene ontologies. (**A**) Loci with an annotated in multicellular organismal development (GO:0007275) are more likely than the whole genome to start paused and have a greater number of loci (15.4% vs 11.9% overall) transitioning state. (**B**) Loci annotated for translation elongation (GO:0006414), a housekeeping function, tend to start and end as active, with no loci changing state. (**C**) Loci annotated for being in the ATP generating proton-pump (GO:0046961), another housekeeping function, also are greatly enriched for those starting and ending as active. A small number of loci (5.3% in this ontogeny) are transcriptionally activating from paused to active. (**D**) Loci annotated as regulators of heart contraction (GO:0008016), are highly enriched for those starting paused, with loci transcriptionally activating or priming for mesodermal or later productive transcription. (**E**) Loci annotated for neurotransmitter receptor activity (GO:0030594) also tend to start paused, but those loci transitioning are instead archiving to silent, reflecting a commitment away from ectodermal lineages, such as neurons. (**F**) Loci annotated for keratinization (GO:0031424) also tend to start paused, with those transitioning archiving to silent, reflecting a commitment away from ectodermal lineages, such as skin.

To better understand the physiological significance of these transcriptional changes from embryonic stem cells to mesoderm, the Gene Ontology database [27] was used to categorize protein-coding loci by annotated function. Compared to the set of all genes, loci involved in multicellular organismal development had a much higher fraction that were transcriptionally paused in embryonic stem cells, with a portion of these either proceeding to active transcription or being archived to a silent state during differentiation (Fig. 4A). Functional categories described as having housekeeping functions, such as translational elongation (Fig. 4B), or components of the ATP-generating proton pump (Fig. 4C) had a much greater fraction of active loci in pluripotent cells, and few of these loci changed state during differentiation. Loci annotated for functions in later mesodermal derivatives, like regulation of heart contraction (Fig. 4D) had a high percentage that were transcriptionally paused during pluripotency and were transcriptionally activated in mesoderm. Conversely, loci annotated for functionality in ectodermal derivatives such as neurotransmitter receptor activity (Fig. 4E) or keratinization (Fig. 4F) tended to start as paused in pluripotent cells, and then were archived to a silent state in mesoderm. This is consistent with ectodermal derivatives being strongly suppressed in our directed differentiation system. Significantly, ontologies with developmentally relevant functions were more likely to contain paused genes becoming both active and silent during differentiation.

We hypothesized that, by focusing on genes that were transcriptionally paused in embryonic stem cells and changed state during commitment to mesoderm, we could predict the later cardiomyocyte fate of the population (Fig S2). Consistent with the future fate of the mesodermal cell population, genes annotated for ectodermal ontologies of neurotransmitter receptor activity, keratinization and brain development have high percentages that lose initiation and are thus archived away. Conversely, the loci of genes in the cardiovascular mesoderm ontologies of heart development, blood vessel development, heart looping and regulation of heart contraction have high percentages that proceed to full-length transcription. By observing how loci exit from paused transcription early in differentiation, we get a strong prediction of future cell fate commitment, one that would not be available through conventional array analysis alone.

## Discussion

**Figure 5:**
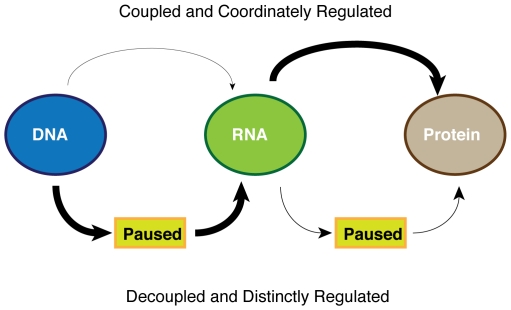
A model for gene regulation during ESC differentiation. The vast majority of genes being activated to transcribe mRNA during ESC differentiation move through the transcriptionally paused state en route to full length mRNA production. Our previous work has shown that once mRNA is synthesized, the majority of transcripts proceed directly to translation at the ribosome, whereas a minority are sequestered from the ribosome (a pause mechanism) and their expression is controlled at the translational level [31].

The paused state of transcription seems to play a central role in the change of gene expression during the differentiation of pluripotent human embryonic stem cells (Fig. 5). By using a directed differentiation system, along with closely spaced temporal resolution, here we were able to observe that transcriptional initiation without elongation is a key transition state for loci undergoing activation or silencing. In our system, as cells commit to mesodermal lineages from pluripotency, we demonstrated that loci changing state seldom gain or lose initiation and elongation together. Rather, these two steps are decoupled, and apparently distinctly regulated. A small subset (∼1%) of genes appeared to move directly from fully active to fully silent and vice-versa, but it is possible that these genes moved through the paused state more quickly than our 2-day resolution could detect.

Several studies have recently shed light into the molecular regulation of transcriptional pausing. The negative elongation factor, NELF, is a four-subunit complex that cooperates with the two-subunit DRB-sensitivity inducing factor, DSIF, to bind PolII and repress elongation [28]. P-TEFb is a protein kinase complex consisting of Cdk9 and Cyclin T [29]. P-TEFb can 1) phosphorylate NELF and promote its dissociation from PolII; 2) phosphorylate DSIF and turn it into a transcriptional activator; and 3) phosphorylate PolII on Ser2 and promote elongation. P-TEFb is thought to move quickly into promoter sites, e.g. in response to histone modifications or binding of specific transcription factors such as c-Myc. Recent ChIP-seq studies indicate that c-Myc binding is a key regulator of RNA synthesis by promoting PolII elongation in multiple genes [30].

The widespread use of transcriptional pausing as a means to control mRNA expression differs significantly from translational regulation during ESC differentiation. We recently performed translation state array analysis on differentiating murine ESCs, and we found that the majority of mRNA transcripts synthesized are loaded directly onto ribosomes [31]. A minority of transcripts (∼10%) are controlled at the level of translation during differentiation (Fig. 5), with mRNAs held in a paused state in pluripotent cells and then loaded with ribosomes. These data indicate that the checkpoint between transcriptional initiation and elongation may represent the major control point for gene expression during ESC differentiation.

The induction of mesoderm requires coordinated signaling through members of the TGF-beta superfamily and the Wnt/β-catenin pathways [14], [32], [33], [34]. Both pathways are known to be strong regulators of transcription, but their contributions to the independent regulation of initiation and elongation remains to be addressed. Further ChIP-chip or ChIP-seq studies for the downstream effectors of these pathways (SMADS and beta-catenin) would be illustrative.

While our study has focused on differentiation of hESCs, we hypothesize that the checkpoint of paused transcription is a more general phenomenon as cells undergo other changes of state. For example, activation of endothelium by cytokines, conversion of blood monocytes to macrophages or differentiation of skeletal myoblasts to myotubes all are accompanied by changes in mRNA transcription. It will be of interest to determine whether paused transcription serves as an obligate checkpoint for transcriptional regulation as these cells change state in response to environmental cues.

## Supporting Information

Figure S1 **Computational analysis of data.** (**A**) To call if a locus is associated with tri-methylation on lysine 4 of histone H3 (H3K4me3), we used the genome-wide chromatin immunoprecipitation data in metagene format, normalized to the transcription start site of each protein coding locus and as a log_2_ over the pan-histone H3 signal at the equivalent probe. The locus was declared H3K4me3 bound if any point within the locus's metagene was above 1.5 log_2_ or over pan-histone H3, over 0.8 log_2_ over pan-histone H3 from −1250 to +750 from the transcription start site. (**B**) The logic flowchart to call each locus as being active (A), paused (P), or silent (S) based on the prior calls for H3K4me3 and full-length transcription for each locus. (**C**) The logic flowchart to determine the transition of each locus from pluripotency to mesendoderm.
(TIF)

Figure S2 **During cardiomyocyte differentiation, ontologies of genes involved in cardiac differentiation activate, those involved in ectodermal derivatives silence.** For each ontology, the percentage of changing loci that are transcriptionally archiving (from initiating to silenced) or activating (from initiating to elongating) is depicted for our differentiation towards cardiomyocytes. The loci of genes in the ectodermal ontologies of neurotransmitter receptor activity (GO:0030594), keratinization (GO:0031424) and brain development (GO:0007420) starting as initiating tend to archive and lose initiation. The loci of genes in the cardiac mesoderm ontologies of heart development (GO:0007507), blood vessel development (GO:0001568), heart looping (GO:0001947) and regulation of heart contraction (GO:0008016) tend to activate and start elongating.
(TIF)
