# Supplementary figures and images for: Evidence That Gene Activation and Silencing during Stem Cell Differentiation Requires a Transcriptionally Paused Intermediate State
Source: PLoS One. 2011 Aug 19;6(8):e22416. doi: 10.1371/journal.pone.0022416 (PMC3158746; doi:10.1371/journal.pone.0022416)

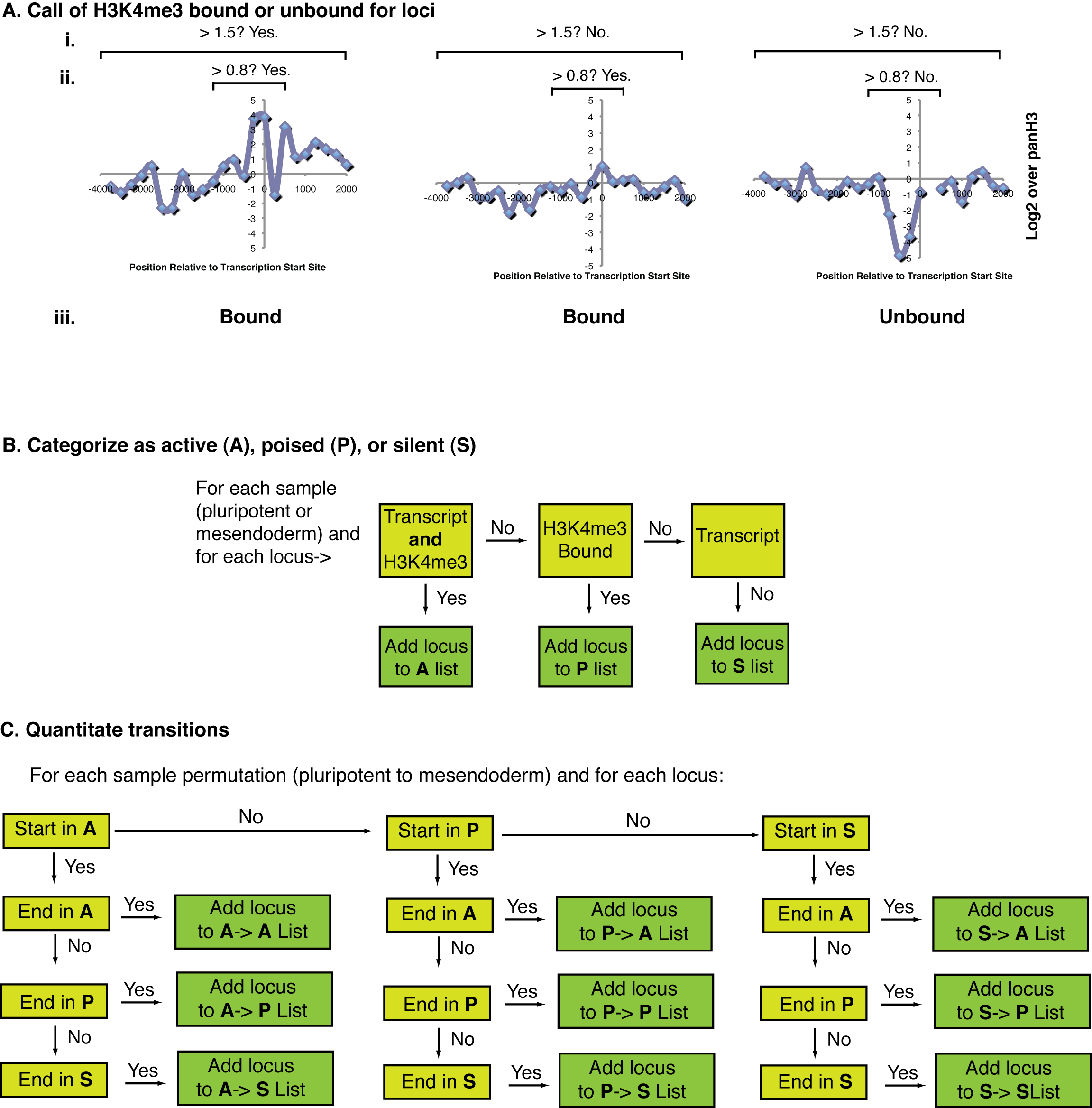

Supplement: Figure S1 — Computational analysis of data. (A) To call if a locus is associated with tri-methylation on lysine 4 of histone H3 (H3K4me3), we used the genome-wide chromatin immunoprecipitation data in metagene format, normalized to the transcription start site of each protein coding locus and as a log2 over the pan-histone H3 signal at the equivalent probe. The locus was declared H3K4me3 bound if any point within the locus's metagene was above 1.5 log2 or over pan-histone H3, over 0.8 log2 over pan-histone H3 from −1250 to +750 from the transcription start site. (B) The logic flowchart to call each locus as being active (A), paused (P), or silent (S) based on the prior calls for H3K4me3 and full-length transcription for each locus. (C) The logic flowchart to determine the transition of each locus from pluripotency to mesendoderm. (TIF) [file pone.0022416.s001.tif]

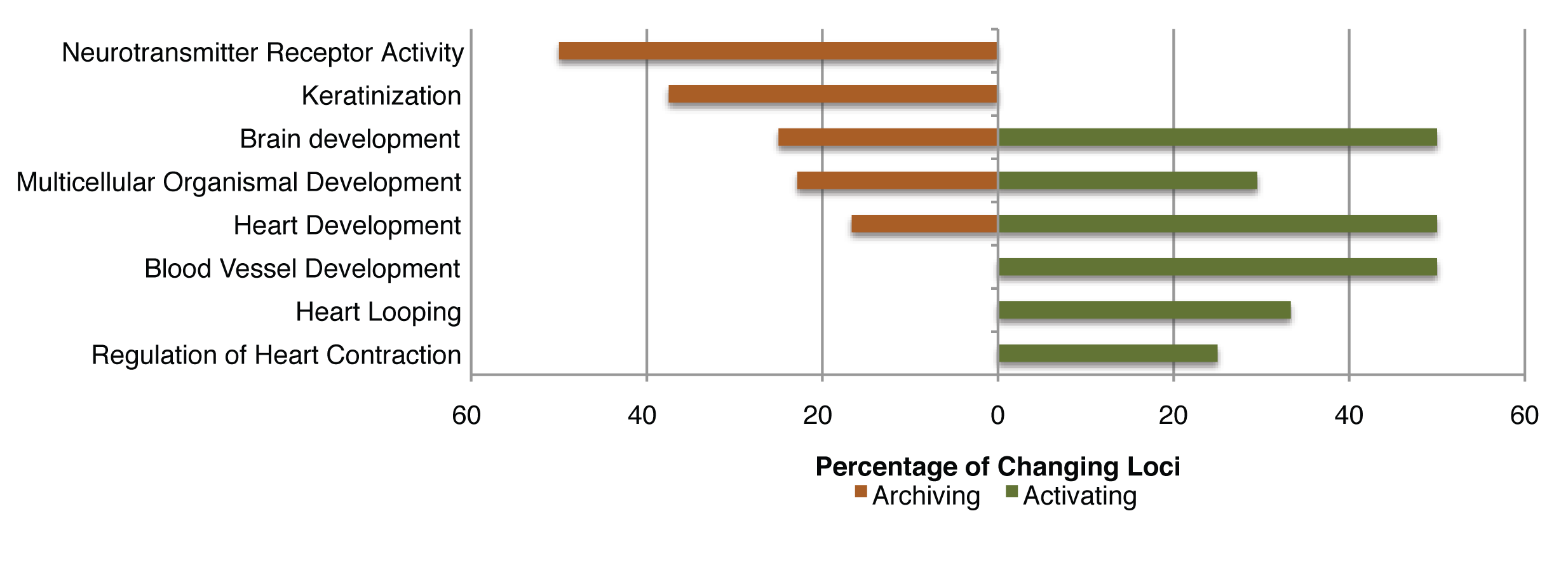

Supplement: Figure S2 — During cardiomyocyte differentiation, ontologies of genes involved in cardiac differentiation activate, those involved in ectodermal derivatives silence. For each ontology, the percentage of changing loci that are transcriptionally archiving (from initiating to silenced) or activating (from initiating to elongating) is depicted for our differentiation towards cardiomyocytes. The loci of genes in the ectodermal ontologies of neurotransmitter receptor activity (GO:0030594), keratinization (GO:0031424) and brain development (GO:0007420) starting as initiating tend to archive and lose initiation. The loci of genes in the cardiac mesoderm ontologies of heart development (GO:0007507), blood vessel development (GO:0001568), heart looping (GO:0001947) and regulation of heart contraction (GO:0008016) tend to activate and start elongating. (TIF) [file pone.0022416.s002.tif]
